# Jaw tone dynamics across varying depths of anesthesia in a swine model

**DOI:** 10.3389/fvets.2026.1858833

**Published:** 2026-07-13

**Authors:** McKenna Andelin, Eduardo Hatschbach, Simone Scherer, Alonso G. P. Guedes

**Affiliations:** Department of Veterinary Clinical Sciences, College of Veterinary Medicine, University of Minnesota, Saint Paul, MN, United States

**Keywords:** anesthetic depth, anesthetic monitoring, isoflurane, nociception, pigs, surgery

## Abstract

**Objective:**

To determine if jaw tone measured objectively can predict a non-surgical depth of anesthesia (DoA) or reliably distinguish between surgical and non-surgical DoA.

**Study design:**

Prospective, randomized, crossover experimental trial.

**Animals:**

Nine, five-month-old, male intact Hanford minipigs.

**Methods:**

A custom device was developed (*n* = 2 pigs) and used to obtain objective measurements of jaw tone at surgical and non-surgical DoA (*n* = 7 pigs), defined according to positive withdrawal to dewclaw clamping during isoflurane anesthesia. Jaw opening force and compliance were analyzed using two-way ANOVA. Areas under curves (AUCs) for jaw opening force and compliance were calculated and compared between DoAs using a two-tailed Wilcoxon matched-pairs signed rank test (*p* < 0.05).

**Results:**

Jaw tone did not change in advance of a positive withdrawal to dewclaw clamping in any of the pigs and therefore was not predictive of non-surgical DoA. There was a minimal and non-significant role of DoA in jaw opening force (2%, *p* = 0.1827) and compliance (0.2%, *p* = 0.9910). Median AUCs for jaw-opening force differed significantly between DoA (*p* = 0.0469), whereas AUCs for compliance did not (*p* = 0.1562).

**Conclusion:**

Jaw tone was not predictive of non-surgical DoA, and did not reliably distinguish between DoA, despite a statistically significant difference in AUC force.

**Clinical relevance:**

Jaw tone is an insufficient predictor of non-surgical DoA and has limited usefulness in distinguishing between surgical from non-surgical DoA.

## 1 Introduction

Accurate assessment of depth of anesthesia (DoA) is central to the clinical application of anesthesiology. Insufficient DoA is a hazard to the patient with deleterious consequences, including stress response, awareness of noxious input, and spontaneous movement that precludes the surgical procedure (1). The 2025 anesthesia and sedation monitoring guidelines published by the American College of Veterinary Anesthesia and Analgesia (2) state that, at a minimum, “a dedicated anesthetist should repeatedly observe the animal to assess eye position, muscle tone, including jaw muscle tone, and reflexes, including the palpebral reflex or peripheral reflexes such as withdrawal of a limb” to evaluate DoA. The introduction of objective measures to help assess DoA is expected to improve anesthetic care and safety, because assessment and interpretation of muscle tone and other subjective DoA indicators may be influenced by the skill and experience of the anesthetist, anesthetic drug protocol, and access to the animal during the procedure (1, 3).

Jaw tone can be described as the baseline tension in the jaw musculature. Jaw tone is assessed by subjectively evaluating the force required for opening the mouth, which is opposed by the muscles that close the jaw. In pigs, similar to most mammals, the muscles that close the jaw include the masseter, temporalis, medial pterygoid, and zygomatico-mandibular muscles (4). The zygomatico-mandibular muscle is absent in humans but present in other domestic veterinary species such as dogs (5) and cats (6). Afferent sensory input is provided by sensory neurons within the mesencephalic nucleus of the trigeminal nerve, which are unique in that their cell bodies are within the central nervous system and not in the trigeminal ganglion. The trigeminal motor nucleus provides efferent output to lower motor neurons, which are responsible for the muscle contraction/tone that underlies jaw tone (7). These processes are moderated by upper motor neurons receiving input from the motor cortex and peripheral afferent input originating from intraoral structures as well as stretch sensory receptors in jaw closing muscles (8). The stretch receptors present in the jaw closing muscles, when activated, produce a monosynaptic supraspinal reflex termed the “jaw-jerk” reflex, masseter reflex, or mandibular reflex, which results in reflexive closing of the jaw (9).

To the author’s knowledge, there are no published studies objectively evaluating jaw tone in anesthetized animals as it relates to DoA. The primary objective of this study was to determine whether jaw tone, measured dynamically with progressive jaw opening, differs significantly between surgical and non-surgical DoA. A secondary objective was to determine whether jaw tone, measured statically, would increase in advance of a positive withdrawal response to a sustained noxious stimulus and therefore be predictive of a non-surgical DoA. We hypothesized that jaw tone would be significantly higher in non-surgical than in surgical DoA, and that it would predict non-surgical DoA by increasing in advance of a positive withdrawal response to a sustained noxious stimulus.

## 2 Materials and methods

### 2.1 Animals

A group of 9 young-adult (5-month-old) sexually intact male Hanford minipigs weighing 17.6 ± 3.7 kg (mean ± SD; Sinclair BioResources, Auxvasse, MO) was included in this study, which was performed concurrently with an unrelated terminal experiment under approval by the University of Minnesota Institutional Animal Care and Use Committee. Aside from vascular catheterization and intrathecal catheter placement at the foramen magnum, which occurred at least two hours prior to any jaw tone assessment, no noxious stimuli were applied as part of the unrelated terminal experiment.

Pigs were included if they were deemed healthy based on physical examination, complete blood cell count, and biochemistry analysis, and were undergoing general anesthesia without significant noxious stimuli. Exclusion criteria included evidence of lesions in the oral cavity, abnormal jaw conformation, and facial asymmetry.

The pigs were group-housed at 3 pigs/pen under controlled environmental conditions with a 12:12-h light–dark cycle and continuous access to water. The animals were allowed a minimum 5-day acclimatization period to the environment prior to any experimental procedures. Pigs were fed twice daily according to a standardized feeding protocol with diet adjusted based on body weight (assessed every two weeks). From weaning until approximately 11 kg, animals received VitaPlus First Feed; 11–22 kg, VitaPlus SureStart (VitaPlus Corporation, WI, USA); and above 22 kg, Envigo/Teklad 7,200 diet (Inotiv, Inc., IN, USA). Diet transitions were performed gradually over a one-week period to minimize dietary stress. Housing, husbandry, and feeding practices were standardized across all animals to minimize variability in growth, behavior, and musculoskeletal development, including jaw musculature, prior to study participation.

### 2.2 Device construction for jaw tone measurement

**Figure 1:**
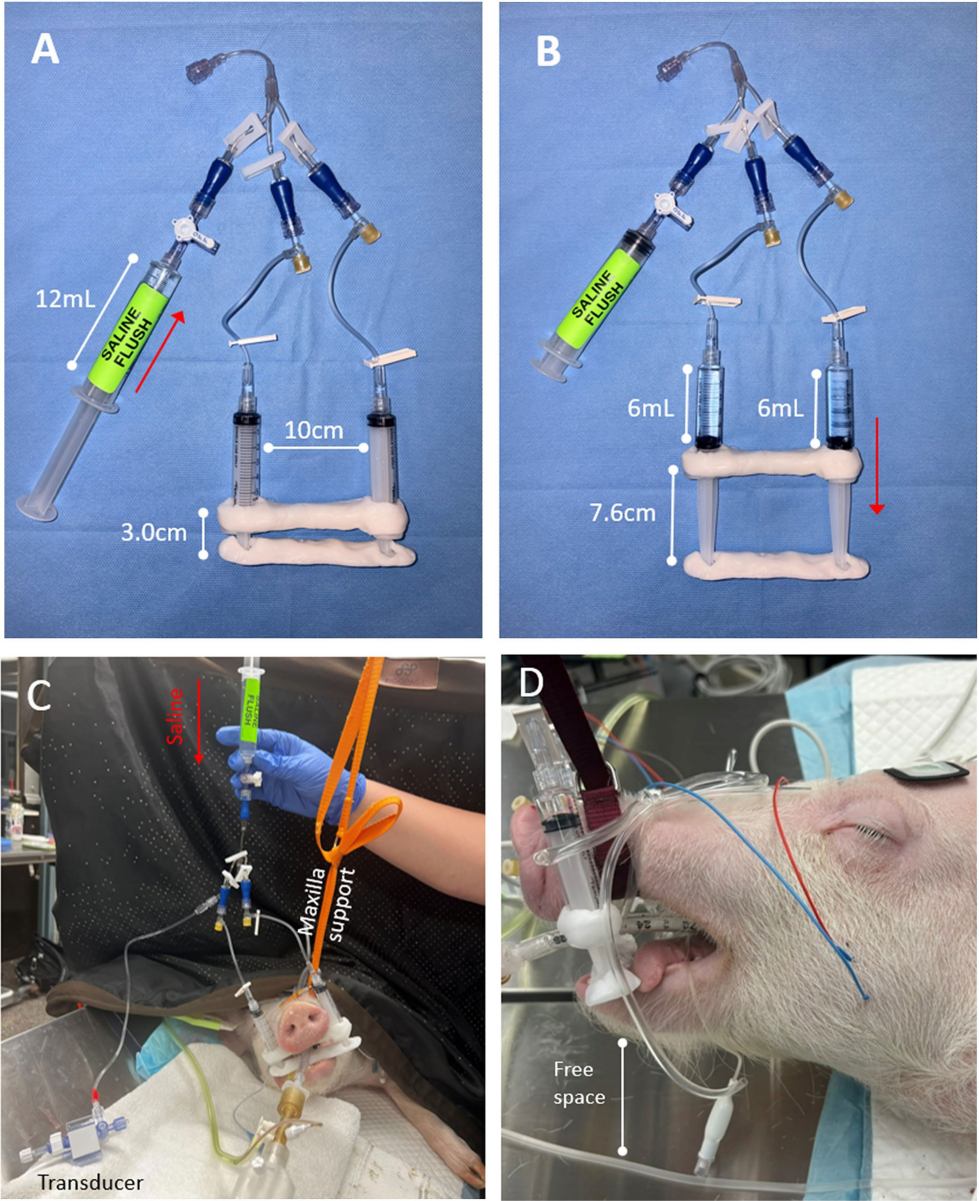
Custom-built device developed to assess jaw tone. **(A)** Device with a 12 mL syringe filled with saline prior to injection. **(B)** Device with the 12 mL syringe emptied and both 6 mL syringes filled with saline after injection, resulting in a device opening of 7.6 cm; the red arrow indicates fluid flow. **(C)** Frontal view of the device positioned in the pig’s mouth, connected to a pressure transducer, with the maxilla suspended. **(D)** Lateral view of the device positioned in the pig’s mouth, showing free space for jaw movement.

A custom-built oral speculum was developed (Figure 1) to enable opening of the mouth and objective measurement of jaw tone. The device was constructed with two 6-mL Luer-lock syringes (Terumo Medical Corporation North America, NJ, USA) by attaching their plungers and barrels adjacent to the flanges using thermoplastic material (Adapt-It thermoplastic pellets, Radiation Products Design Inc., MN, USA). The thermoplastic formed two rigid bars connecting these two syringes, with a 10 cm gap between them to accommodate the width of the pig’s snout. The 6-mL syringes were then connected to the female ports of two Luer-lock T-connector short extension sets (B. Braun Medical Inc., PA, USA), which were subsequently connected to the female ports of the tri-port extension set (ICU Medical Inc., CA, USA). The third female port of the tri-port was connected to a 3-way stopcock and 12-mL syringe (Monoject™, Cardinal Health, OH, USA). The male terminal of the tri-port extension set was connected to a pressure transducer (Deltran®, Utah Medical Products Inc., UT, USA) via 12-inch-long non-compliant tubing. The transducer calibration was verified before each experiment using a water column with heights ranging from 0–250 cm (corresponding to 0–250 cm H2O) and transducer measurements were compared against the reference water-column (10 cm H2O = 7.36 mmHg). Per the manufacturer, the transducers have an accuracy ± 2% or 1 mmHg, whichever is greater, with drift of less than ± 1 mmHg over an 8-h period. The transducer was zeroed to atmospheric air at the level of the mandible. The 12-mL syringe and connecting lines were filled with fluid (0.9% sterile saline) and freed from any air, and this fluid was used for filling the two 6-mL syringes of the device to actuate jaw opening. The speculum was positioned intraorally such that the thermoplastic bars linking the plungers contacted the mandible, while the bar connecting the flanges of the syringe barrel contacted the maxilla. With this configuration, the thermoplastic bar connecting the plungers of the 6-mL syringes opened the mandible when fluid from the 12-mL syringe was rapidly introduced, thereby mimicking the jaw-opening motion used by the anesthetist to assess jaw tone. Two pigs were utilized during prototype development. Notable outcome measures from the device construction were the opening provided by the device at minimum (completely closed, with room to accommodate an endotracheal tube) and at maximum (completely opened).

Linearity of the system was confirmed under dynamic and static conditions to ensure that non-linear behavior of the hydraulic-mechanical system did not obscure true biological differences. To test under dynamic conditions, the system was affixed to a flat surface and assessed under dynamic conditions by contacting the thermoplastic bar that contacts the mandible to the plunger of an air-filled 60-ml glass syringe, which was attached to a piece of flexible silicone tubing and a sphygmomanometer. When the device was actuated, it pushed against the glass syringe’s plunger, displacing air into the silicone tubing and the sphygmomanometer, mimicking dynamic mandibular opening. To test under static conditions, the device was affixed upright, with the thermoplastic bar that opens the mandible facing up. Loads of 0.5, 1, 2, and 4 kg were applied to the device at the minimum and maximum possible opening distances, mimicking mandibular pressure applied to a static device. The resulting pressures under both conditions were recorded with the transducer and were found to be linear.

### 2.3 Anesthesia and determination of anesthetic depth

General anesthesia was induced and maintained using isoflurane delivered in 100% oxygen, administered via face mask and orotracheal tube, respectively. Each pig was positioned in sternal recumbency, and the head was gently suspended by a dog leash looped around their maxilla to allow free jaw movement. The orotracheal tube remained centered within the custom-built speculum (Figure 1C) and the rebreathing circuit and associated instrumentation were supported to maintain free mandibular movement without interference from the weight of the system. Intermittent positive pressure ventilation was instituted to obtain near normal end-tidal carbon dioxide (ETCO_2_; 45–55 mmHg) using a mechanical ventilator (Hallowell Veterinary Anesthesia Ventilator, Hallowell EMC, MA, USA). Physiologic and anesthetic parameters collected for this study included ETCO_2_ and isoflurane (ET_ISO_) tensions by sidestream capnography (Datex-Ohmeda Compact S5 - GE Healthcare, Chicago, IL, USA). Other physiologic parameters monitored included heart rate via a 3-lead ECG, mean arterial blood pressure obtained via arterial catheterization of the saphenous artery. Presence or absence of limb withdrawal in response to a standardized noxious stimulus was used to classify DoA as non-surgical or surgical, respectively. The stimulus consisted of applying a 15-cm curved Doyen forceps to a pelvic limb dewclaw and closing it to the first ratchet for up to 60 s or until a positive response was elicited, whichever occurred first. A positive response was defined as a withdrawal response or any observable gross purposeful movement occurring during the 60-s stimulation period. The jaws of the forceps were covered by tight-fitting plastic tubing to minimize tissue trauma. ET_iso_ concentrations were adjusted in small increments (~0.25%), allowing for a 5-min equilibration period at each increment, until the desired DoA was achieved. No blinding was implemented during the study.

### 2.4 Data collection

#### 2.4.1 Static measurements

The goal of these measurements was to determine if jaw tone increases before a positive response to a painful stimulus and therefore can be used to predict a non-surgical DoA. With the jaw slightly opened in a static position to a stable pressure of ~100 mmHg using the custom-built device, dewclaw clamping was applied and the jaw tone was recorded continuously using a PowerLab C data acquisition device (ADInstruments, RRID: SCR_028296). This was done under both non-surgical and surgical DoA, although the main interest was in the non-surgical state. The primary outcome measure for the static measurements was an increase in jaw tone during noxious stimulus, recorded as a binary variable (present or absent). The response latency was also recorded for positive responses.

#### 2.4.2 Dynamic measurements

The goal of these measurements was to determine if jaw tone can reliably distinguish between known surgical and non-surgical DoA. Once the desired DoA was achieved, a series of progressively larger jaw openings was performed using the custom-built device. Jaw opening was accomplished by rapid manual infusion of saline, causing the device to open the jaw to predetermined distances. Infusions were delivered in 1-mL increments, up to a total volume of 12 mL. To accommodate both the device and the orotracheal tube within the center of the speculum, the jaw was opened 3 cm, and each mL infused resulted in additional jaw openings of 0.38 cm, with a maximum jaw opening of 7.6 cm. Measurements were performed in triplicate at each volume/distance, and the resulting pressures were recorded with a PowerLab C data acquisition device (AD Instruments, RRID: SCR_028296). Upon completion of each measurement series, DoA was reassessed to confirm that the animal remained at the intended DoA throughout data collection. Only measurements in which DoA was unchanged before and after data acquisition were included in the analysis. The measurements were then repeated in the same manner at the alternate DoA. The sequence of DoA was randomized (random.org). The primary outcome measure for the dynamic measurements was the pressure measured by the custom-built device in mmHg. These measurements were used to calculate jaw opening force and compliance (see below).

After data collection, pigs were euthanized via intravenous injection of pentobarbital sodium. Death was confirmed by the absence of arterial and ECG waveforms, audible heart sounds, and corneal reflex. Dynamic jaw tone measurements were collected after euthanasia in 3 pigs to gain insight into jaw tone at what could be considered excessive DoA for comparison. These data are presented descriptively and were not included in the statistical analysis.

### 2.5 Statistics

As there is no published data on objective measurement of jaw tone in any species under general anesthesia (to the authors’ knowledge), necessary information for an *a priori* sample size calculation was unavailable. Thus, the sample size was based on the availability of eligible pigs.

The pressure values were converted to force, using the conversion factor 1 mmHg = 1.3332239 N/cm^2^, before statistical analysis in GraphPad Prism Version 10.6.1 for MAC OS (RRID: SCR_002798). Normality was assessed by visual inspection of QQ plots and the Shapiro–Wilk test. Jaw opening compliance was calculated as the jaw opening distance/force to assess how easily the jaw muscles stretch in response to force across different DoA. The jaw opening forces and compliances were analyzed using two-way ANOVA with multiple comparisons corrected using statistical hypothesis testing, with force, compliance, and DoA as fixed effects and pig as a random effect. Areas under the curve (AUC) for jaw opening force and compliance were calculated using the trapezoidal method to assess the integrated effort to fully open the jaw. These AUCs and ET_ISO_ for the two DoA were not normally distributed and were compared using a two-tailed Wilcoxon matched-pairs signed rank test. Confidence intervals for the median paired differences in AUC for force and compliance were calculated. Significance level was set at *p* < 0.05 and was not adjusted for multiple comparisons. Data are shown as mean±SD or as median±IQR and individual values.

## 3 Results

### 3.1 Prototype development and anesthetic variables

All 9 pigs available for the study met the inclusion/exclusion criteria and were included. Two of the pigs were used to develop and refine the custom-built device, yielding a complete data set with no missing values from 7 pigs at both DoA with the final prototype. The minimum opening of the jaw with the speculum was 3 cm, which was necessary to accommodate the endotracheal tube. Each mL of saline infused resulted in an additional 0.38 cm opening, for a total jaw opening of 7.6 cm when the full 12 mL were infused.

For the non-surgical and surgical DoA, respectively, the median (IQR) ETCO2 was 44 (43, 47) and 47 (45, 52) mmHg, heart rate was 137 (122, 171) and 147 (122, 171) beats/min, and mean arterial pressure was 86 (78, 103) and 91 (72, 96) mmHg. These variables were not compared statistically. Median (IQR) ETISO was 1.85% (1.6, 2.35) and 1.4% (1.4, 1.9) for the surgical and non-surgical DoA, respectively, and the difference was statistically significant (*p* = 0.0313).

### 3.2 Static measurements

**Figure 2:**
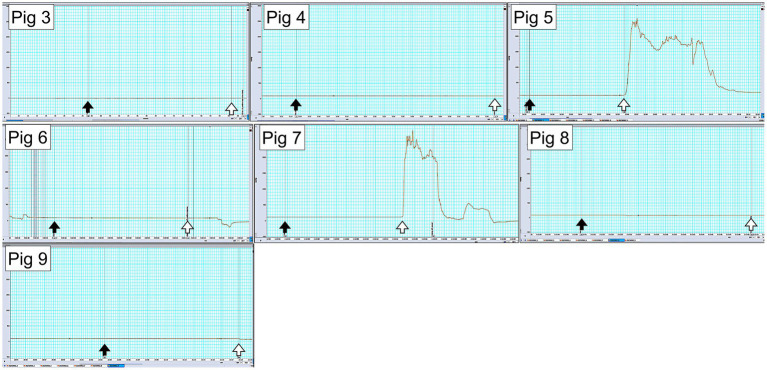
Static measurements of jaw tone during application of noxious stimulus under a non-surgical depth of anesthesia. Pressure is shown on the y-axis (range: −500 to 3,000 mmHg across all panels), and time is on the x-axis in seconds (all panels display the 60 s intended for noxious stimulation). The jaw was partially opened using a custom-built device to a stable pressure (~100 mmHg) before application of a noxious stimulus. The noxious stimulus was produced with a 15-cm Doyen forceps applied to a dewclaw and closed to the first ratchet (black arrow) for 60 s or until a limb withdrawal response was observed, whichever came first (white arrow). All pigs had positive limb withdrawal, no pigs had changes in jaw tone leading up to limb withdrawal and only 2 pigs (pig 5 and pig 7) had an abrupt and marked increase in jaw tone concurrently with limb withdrawal.

The results are illustrated in Figure 2. The median (range) response latency (i.e., time from dewclaw clamp application to a positive limb withdrawal) was 21 (11, 34) seconds. Jaw tone remained stable and did not change in advance of a positive reaction to dewclaw clamping in any of the pigs under non-surgical DoA. In only 2/7 pigs (pigs 5 and 7), there was an abrupt, marked increase in jaw tone concurrent with a positive response to the dewclaw clamp, but jaw tone remained stable up to this point. At surgical DoA, no changes in jaw tone occurred during clamp application (data not shown).

### 3.3 Dynamic measurements

**Figure 3:**
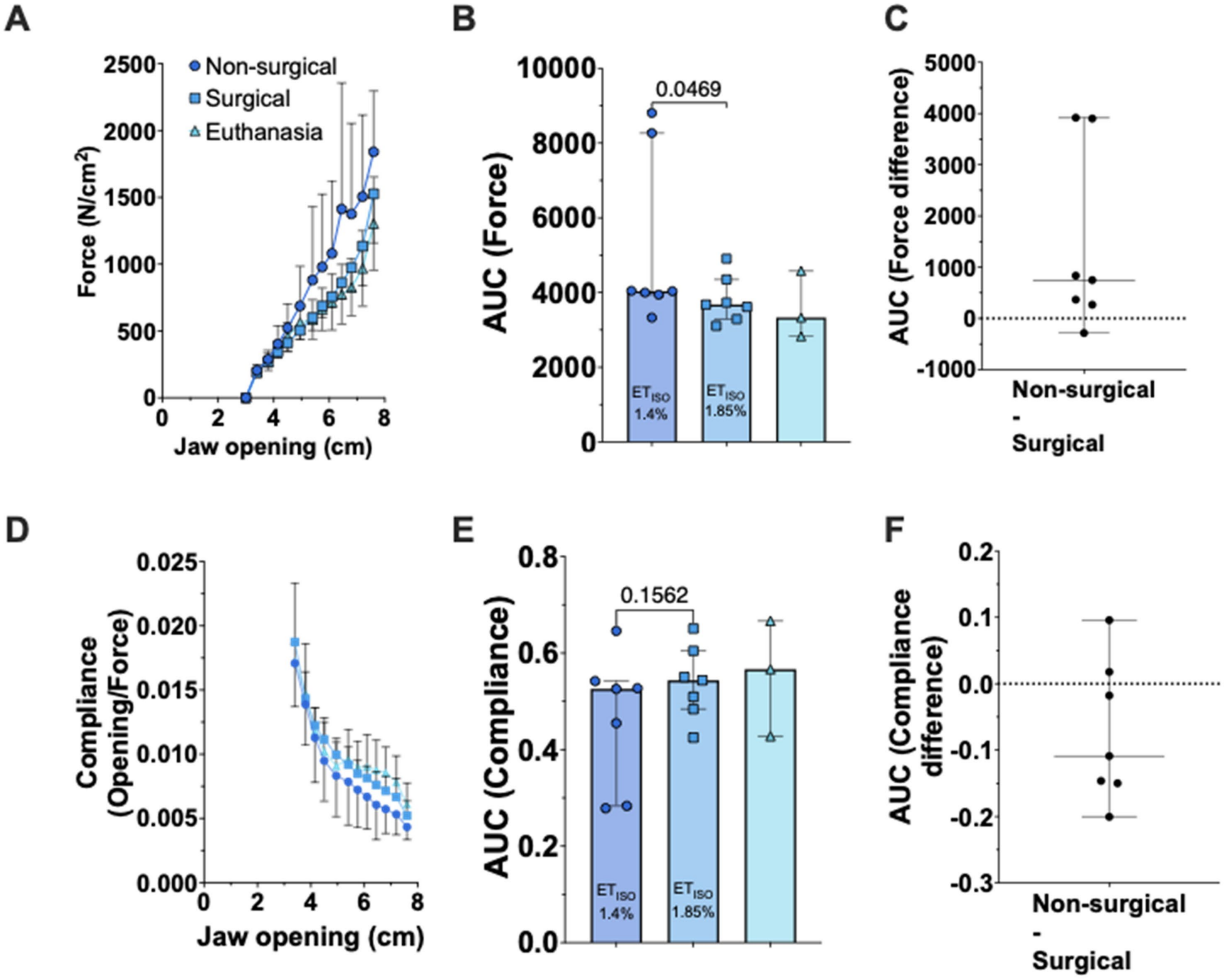
Results of dynamic assessment of jaw tone during non-surgical and surgical depths of anesthesia (DoA) in isoflurane-anesthetized Hanford minipigs (*n* = 7). Jaw opening force **(A)**, area under the curve (AUC) for force **(B)**, difference plot for AUC force **(C)**, jaw opening compliance **(D)**, AUC compliance **(E)**, and difference plot for AUC compliance **(F)**. Measurements were also obtained in a subgroup of *n* = 3 pigs after euthanasia with pentobarbital overdose for visual comparison. The DoA was defined as non-surgical and surgical, respectively, by the presence or absence of limb withdrawal in response to the application of a dewclaw clamp with a 15-cm Doyen forceps closed to the first ratchet. The median end-tidal isoflurane concentration at each anesthetic depth is indicated within the bars in **(B,E)**. The AUC force and AUC compliance were compared using a two-tailed Wilcoxon matched-pairs signed rank test, with *p* < 0.05 considered statistically significant. Data are shown as mean±SD **(A,D)** or as median±IQR and individual values **(B,C,E,F)**.

The results of jaw opening forces and compliances (mean±SD) calculated from the pressure readings obtained with the device, the respective calculated AUC (median±IQR), and difference plots are shown in Figure 3. Visual inspection of the jaw opening force (Figure 3A) and compliance (Figure 3D) curves shows increasing separation between the means for the two DoA, starting at approximately 5 cm of jaw opening, which corresponds to approximately 65% of the maximum (7.6 cm). This separation was more evident in measurements obtained after euthanasia, especially for compliance, which likely represents maximal anesthetic-induced muscle relaxation. However, there was marked variability in the results and overlapping standard deviations across all groups, such that the interaction between jaw opening force and DoA explained only 2% of the total variation and was not statistically significant (*p* = 0.1827). Progressive jaw opening, regardless of DoA, accounted for the largest proportion of the total variation (64%; *p* < 0.0001), followed by pig (18%; *p* < 0.0001) and DoA (4%; *p* = 0.1469). Similarly, for jaw opening compliance, the interaction between compliance and DoA explained only 0.2% of the total variation and was not statistically significant (*p* = 0.9910). Progressive jaw opening explained most of the total variation (71%; p < 0.0001), followed by pig (19%; p < 0.0001) and DoA (2%; *p* = 0.2443).

Median AUCs for jaw-opening force (Figure 3B) differed significantly between DoA (*p* = 0.0469; median paired difference 748; 98% CI: −286, 3,918), whereas AUCs for compliance (Figure 3E) did not (*p* = 0.1562; median paired difference −0.11; 98% CI: −0.2, 0.1). Difference plots demonstrated that a greater force was required to open the jaw in most animals (6 out of 7) during non-surgical compared to surgical DoA (Figure 3C). Similarly, jaw compliance was lower in most animals (5 out of 7) during a non-surgical DoA compared to a surgical DoA (Figure 3F). Finally, the median (IQR) coefficients of variation (CV%) for all triplicate dynamic measurements of jaw tone in the non-surgical and surgical DoA were, respectively, 6.4% (5.2, 12.2) and 5.4% (4.2, 10.9), whereas the CV% following euthanasia of the 3 pigs was 6.0% (4.7, 13.7).

## 4 Discussion

Jaw tone assessment is commonly recommended and performed during anesthesia to help assess DoA in many animal species, including pigs (2, 10). The procedure is usually performed by subjectively assessing the degree of difficulty or ease to manually opening the jaw (11, 12). A relaxed, easy to open jaw is interpreted as indicating adequate or surgical DoA, whereas a more tense and difficult to open jaw is interpreted as inadequate DoA for surgical intervention (1, 13). However, no previously published studies have objectively evaluated whether jaw tone can be used to reliably distinguish between non-surgical and surgical DoA, or to anticipate a physical reaction during non-surgical DoA. Hence, the results of this study bridge this knowledge gap. Although the integrated effort to fully open the jaw (AUC force) differed significantly between non-surgical and surgical DoA, the magnitude of this difference was small, and there was no clear separation in the distribution of individual data points. Additionally, the 98% confidence interval for the median of the paired differences was very wide, supporting the limited confidence in this statistically significant result. Further, the relaxation state of the jaw muscles, assessed via AUC compliance, did not differ significantly between non-surgical and surgical DoA. The narrow 98% confidence interval for the median of the paired differences in AUC compliance supports good confidence in this result. In addition, the static measurements showed that jaw tone is a poor predictor of impending physical movement during non-surgical DoA. Thus, despite widely used and assessed subjectively, our results with objective measurements suggest that jaw tone might have limited utility as an indicator of DoA in clinical settings.

Pigs are an important model in biomedical research, commonly undergoing noxious procedures under general anesthesia. Studies aimed at validating and refining DoA evaluation methods in swine are critical for advancing anesthetic management and welfare standards in both clinical and research settings. Recent literature has described the challenges associated with the reliable assessment of DoA in pigs, with no clear consensus on clinical indicators of DoA, including both subjective clinical signs (e.g., motor responses, reflexes, muscular tone) and quantitative measures (e.g., electroencephalogram [EEG] or EEG-based monitoring) (14). In the current study, visual inspection of jaw tone curves (force and compliance) of non-surgical and surgical DoA indicates a separation between the means when jaw opening > 60% of maximum. Furthermore, the difference plots showed that jaw tone was generally higher during non-surgical versus surgical DoA. Anesthesia providers seek to identify these features through repeated clinical evaluations over the course of anesthesia. However, this is a difficult task based on our results using objective measures, which indicated that DoA contributed minimally to changes in jaw tone and the interaction between jaw tone, measured as pressure/force or compliance, and DoA was minimal and not statistically significant. Additionally, jaw tone did not change in any of the pigs in advance of a positive physical response while in non-surgical DoA, further highlighting the limitation of jaw tone to assess non-surgical vs. surgical DoA. It is worth considering that subjective manual force detection by an anesthetist was not performed in this study as the ACVAA guidelines suggest; correlation of these results with manual force detection is an untested element for future research. While jaw tone is a poor standalone indicator of DoA in pigs, it may retain some clinical utility if integrated with other subjective measures, including anesthetic dose, cardiorespiratory responses, and palpebral reflex assessment.

The current study utilized limb withdrawal in response to a noxious stimulus as a clinically relevant marker for non-surgical DoA, unlike traditional experimental methods to determine anesthetic dose that rely on gross purposeful movement (15). The dose of isoflurane required to inhibit nociceptive withdrawal reflex to dewclaw clamping is higher than for preventing gross purposeful movement in pigs (16), which contrasts with the lower dose required to abolish withdrawal reflexes than for preventing gross purposeful movement in humans (17). While spinal reflexes are known to be dose-dependently suppressed by isoflurane anesthesia, as assessed by H-reflex latency (18), making a similar assumption for supraspinal jaw reflexes may be incorrect due to the neuroanatomic differences of the jaw closing muscles. It is possible that using limb withdrawal rather than gross purposeful movement to determine DoA in the current study may have hindered recruitment of upper motor neuron pathways necessary to increase jaw tone (7), representing a central interpretive limitation of the study design. This can also be an important clinical limitation if the anatomical and physiological differences between reflexes are not taken into consideration. Thus, when using jaw tone to assess DoA, species-specific anesthetic sensitivity and differences in jaw-related neuroanatomy, particularly during painful procedures elsewhere in the body, must be considered.

The current study has some additional limitations worth considering. If the true difference in jaw tone between DoA is small, the study may have been underpowered to detect statistically significant differences. Nonetheless, given the minimal interaction observed between jaw tone and DoA, it is unlikely that even statistically significant differences would translate into significant clinical relevance. It is possible but unlikely that the measurement device obscured significant differences in jaw tone because its responses were linear under both dynamic and static conditions. Nonetheless, mechanical losses and device compliance remain potential confounding factors. Furthermore, anesthesia was maintained using isoflurane alone, and results may not be extrapolated to other anesthetics or multimodal protocols that differentially affect neuromuscular function. Although equilibration periods were used following changes in isoflurane concentration, complete tissue equilibration may not have occurred, and minor fluctuations could have obscured subtle changes in jaw tone during dynamic assessment. However, animals were at a reasonably stable DoA during assessment since DoA was confirmed after each measurement session in all animals. The current results in pigs may not be extrapolated to other veterinary-relevant animal species, especially considering the broad anatomical variations between and even within a given species. One feline study where jaw tone was qualitatively assessed, reported a trend toward jaw relaxation with increasing DoA (19). This observation is largely consistent with our findings that jaw tone tends to decrease as DoA increases. However, our objective results suggest that, at least in isoflurane-anesthetized pigs, jaw tone may not reliably distinguish clinically relevant differences in DoA. It is possible that in pigs, significant jaw muscle relaxation occurs before what is defined as non-surgical DoA, with further increases in DoA reflecting minimal additional changes in jaw tone. It is possible that findings in this study with pigs in sternal recumbency would differ in alternative recumbencies (dorsal or lateral) due to changes in head and neck position, gravitational effects on the mandible, the impact of lateral excursion or jaw protraction, and/or changes in the geometry of the temporomandibular joint. Regardless of recumbency, it is important that the device and jaw are unobstructed to ensure free jaw movement. In conclusion, contrary to the hypotheses, the jaw tone did not increase progressively during application of a mechanical noxious stimulus and in advance of physical movement when pigs were at a non-surgical DoA. The effort required to open the jaw was statistically significantly greater in non-surgical compared to surgical DoA, but the difference appeared small and highly variable to be clinically relevant. The jaw muscle relaxation state (i.e., compliance) did not differ significantly between DoA. Taken together, our results when using this porcine model suggest that jaw tone is a weak indicator of DoA. Further studies are needed to determine the utility of jaw tone in other veterinary-relevant animal species.

## Data Availability

The raw data supporting the conclusions of this article will be made available by the authors, without undue reservation.
